# Anti-3-Hydroxy-3-Methylglutaryl-Coenzyme A Reductase Immune-Mediated Necrotizing Myopathy following mRNA SARS-CoV-2 Vaccination

**DOI:** 10.1155/2023/7061783

**Published:** 2023-05-27

**Authors:** Marta Rodrigues de Carvalho, Myrian Mathildes Sá de Deus Rocha, Vinícius Alves Bezerra, Maciel Eduardo de Pontes, Maria Cristina del Negro, Julio Salgado Antunes, Vinícius Viana Abreu Montanaro, Rubens Nelson Morato Fernandez

**Affiliations:** ^1^Department of Neurology and Clinical Neurophysiology, Base Hospital of the Federal District, Brasília, Distrito Federal, Brazil; ^2^Department of Radiology, DFSTAR Hospital, Brasília, Distrito Federal, Brazil; ^3^Department of Neurology, Sarah Network of Rehabilitation Hospitals, Brasília, Distrito Federal, Brazil; ^4^Department of Pathology, Sarah Network of Rehabilitation Hospitals, Brasília, Distrito Federal, Brazil

## Abstract

The new coronavirus (COVID-19) pandemic has resulted in the unprecedented production of vaccines. In this context, the possible adverse effects remain to be identified and reported. In this article, we report the case of a young female patient who developed anti-3-hydroxy-3-methylglutaryl-coenzyme A reductase (anti-HMG-CoA) immune-mediated necrotizing myositis (IMNM) after receiving the Pfizer-BioNTech (BNT162b2) COVID-19 vaccine. The diagnosis of probable post-vaccination IMNM was made due to the absence of other factors that may have led to the development of autoantibodies (medicines; e.g., statins, drugs) and the temporal relationship between exposure and event. This case report is the first to suggest that a COVID-19 vaccine may trigger anti-HMG-CoA reductase necrotizing myopathy.

## 1. Introduction

In 2019, the novel severe acute respiratory syndrome coronavirus 2 (SARS-CoV-2) was identified as responsible for pneumonia of unknown cause in Chinese patients [1, 2]. On March 11, 2020, the World Health Organization classified the global outbreak of the disease as a pandemic [3]. In this setting, several vaccines have been produced and released to the public at unprecedented speeds and quantities, putting the scientific community and the world population on alert for possible adverse events [4]. In this paper, we report a case of immune-mediated necrotizing myositis (IMNM) that developed one week after a patient received the first dose of the COVID-19 vaccine Pfizer-BioNTech (BNT162b2).

## 2. Case Presentation

A 22-year-old woman of African ancestry, with no medical history of previous comorbidities or use of any medication, was admitted to our tertiary hospital with symptoms of marked muscle weakness, loss of ambulation, and elevated creatine kinase levels (21,921 U/mL).

She reported the onset of symptoms one week after receiving the first dose of the Pfizer-BioNTech (BNT162b2) vaccination (applied on August 15, 2021). The initial symptoms were paresthesia in the shoulder girdle and tightness and pain in the upper limbs, mainly when moving and lifting objects. She worked as a clerk in a supermarket; after seeking medical care, the symptoms were attributed to her profession.

The symptoms progressed to weakness of the lower limbs, severe girdle weakness, and loss of walking ability. The patient was admitted to our hospital in December 2021. On admission, her physical examination revealed symmetrical weakness in the neck and extremities, specifically Medical Research Council grade 3/5 in her neck and proximal upper limbs, grade 4/5 in the distal upper limbs, grade 2/5 in her proximal lower limbs, and grade 3/5 in the distal lower limbs. Tendon reflexes of the extremities were normal. No abnormalities were found on cranial nerve testing. No sensory, autonomic, or coordination disturbance was observed.

**Figure 1:**
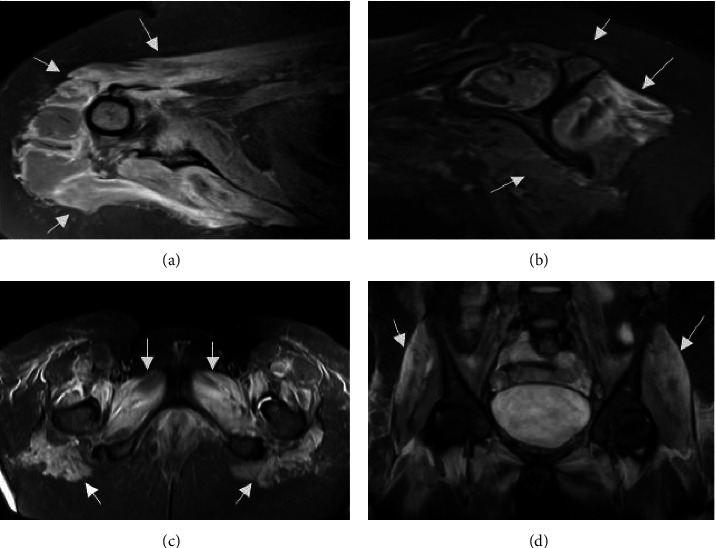
(a) Magnetic resonance imaging (MRI) of the shoulder, oblique axial view, DP sequence, with fat saturation, showing hyperintensity in the m. pectoralis major and m. deltoid. (b) MRI of the shoulder, oblique sagittal view, T2 sequence with fat saturation, showing hyperintensity in the rotator cuff muscles (supraspinatus, infraspinatus, and subscapularis). (c) MRI of the pelvis, axial section, and STIR sequence, showing hypersignal in the adductor and extensor muscles of the thigh and in the gluteus maximus. (d) MRI of the pelvis, coronal section, and STIR sequence, showing hyperintensity in the thigh extensor musculature.

At admission, her blood count and biochemical examinations did not show any alterations except for creatine kinase (21,921 U/L). Serological tests for HIV, HTLV, brucellosis, Chagas disease, toxoplasmosis, and treponema were all negative. Electroneuromyography of the upper and lower limbs showed sensory conduction velocities, amplitudes, and latencies of sensory action potentials of the median, ulnar, sural, and superficial peroneal nerves within normal limits; motor conduction velocities, distal latencies, and amplitudes of compound muscle action potentials of the median, ulnar, tibial, and deep peroneal nerves within normal limits; F waves with preserved latencies in the median, ulnar, tibial, and deep peroneal nerves. Electromyography showed increased activity of insertion, fibrillation potentials, and discharge of acute positive waves at rest in the deltoid, triceps, biceps brachii, pronator teres, first dorsal interosseous, abductor pollicis brevis muscles, trapezius, iliopsoas, quadriceps, tibialis anterior, gastrocnemius medial, and adductor magnus. During the voluntary contraction test, polyphasic action potentials of the motor unit were observed with reduced durations and amplitudes and increased recruitment and interference patterns. Echocardiography and chest and abdominal computed tomography showed no alterations. Magnetic resonance imaging of the shoulder and pelvic girdles was performed, which showed diffuse edema of the musculature (Figure 1).

The patient received methylprednisolone intravenously (1 g per day for five days) and additional symptomatic treatment. She showed mild improvement in motor function. The hypothesis of inflammatory myopathy of post-vaccination etiology was made, and after 10 days of hospitalization, the patient was discharged with a proposal to continue the investigation on an outpatient basis (discharge creatine kinase: 10,931 U/mL).

After hospital discharge, vaccination campaigns were still in force in our country, and the patient inadvertently received the second dose of the Pfizer-BioNTech (BNT162b2) COVID-19 vaccination on January 7, 2022. One week later, she experienced further marked deterioration in motor strength, losing the ability to walk, and requiring a second cycle of intravenous methylprednisolone (1 g per day for five days). In addition, her creatine kinase level was elevated (13,519 U/mL). The patient was then readmitted to the hospital for treatment and was discharged five days later.

After the second admission for treatment with methylprednisolone, during the outpatient investigation, rheumatological tests (antinuclear factor, rheumatoid factor, and antisynthetase antibodies) were performed and resulted in negative.

**Figure 2:**
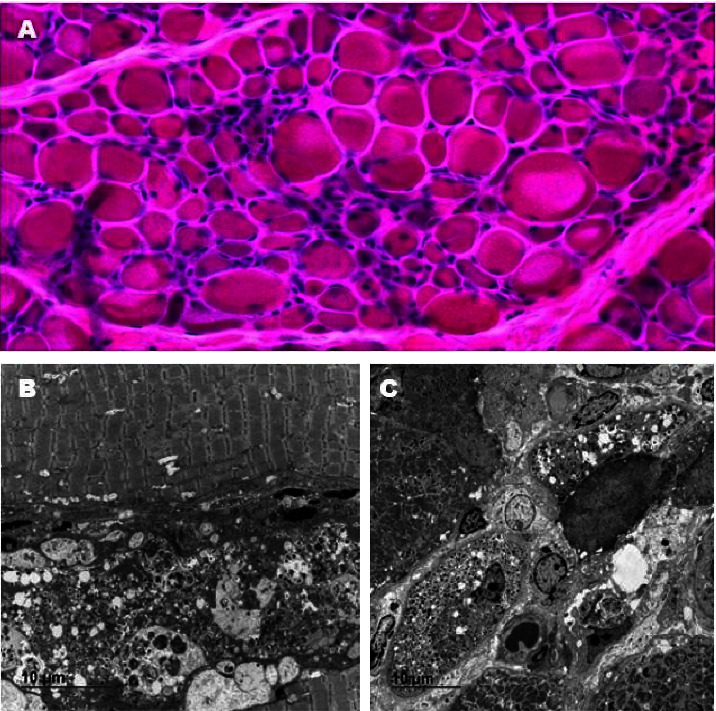
(a) Hematoxylin and eosin analysis shows angular and rounded atrophic fibers, diffuse inflammatory reaction, foci of fiber necrosis, and phagocytosis of necrotic fibers. (b and c) Transmission electron microscopy analysis reveals variation in fiber caliber, presence of necrotic fibers, autophagic voids of emissions filled with dense granules and membrane remnants, subsarcolemmal occurrences of mitochondria, and perivascular and endomysial inflammatory infiltrates.

In order to help in the diagnostic elucidation, a muscle biopsy was performed. ATP 9.4, DPNH, esterase, Gomori, hematoxylin and eosin, PAS, SDH, and Sudan techniques were used. A diffuse inflammatory reaction was observed, with islands of focal necrosis of fibers, phagocytosis of necrotic islands, marked variation in fiber caliber diameter, marked fiber atrophy, with angular and rounded atrophic fibers, moderate endomysial, and perimysial conjunctival proliferation. Immunohistochemistry showed strongly intense positivity for the following markers: CD20; CD3; CD31; CD4; CD56; CD68; CD8; C5b-9; MCH class I; SQSTM1 (P62). Transmission electron microscopy analysis revealed skeletal striated muscle with ultrastructural alterations: fiber caliber variation; frequent necrotic fibers; autophagic vacuoles of varying sizes, which are filled with dense granules and remnants of membranes; subsarcolemmal and intermyofibrillar accumulations of glycogen; lipid drops; eventual subsarcolemmal accumulations of mitochondria; perivascular and endomysial inflammatory infiltrate; fibrosis. Taken together, these findings are suggestive of IMNM (Figure 2).

After the biopsy, a myositis panel analysis was performed. Anti-3-hydroxy-3-methylglutaryl-coenzyme A reductase (anti–HMG-CoA) was positive at titers almost 10 times above the reference value (result: 193 AU; reference value: <20 AU). Antisignal recognition particle (SRP) tests were negative. A genetic panel was done, and one heterozygous variant of the uncertain significance of the dysferlin gene was described as NM_003494.4(DYSF): c.2093C>T (p.Ala698Val). Type 2 girdle muscular dystrophy is a condition of autosomal recessive inheritance, requiring two causative variants in distinct alleles for the complete expression of the clinical picture. The presence of an isolated variant, as in the case described, is not sufficient for the development of the phenotype. Additionally, we performed western blot to assess dysferlin expression in the muscle, which resulted in the normal presence of calpain and dysferlin bands.

We decided to perform immunosuppressive treatment with methotrexate (15 mg/week) and corticosteroids (80 mg/day); however, the patient persisted with severe muscle weakness, high levels of creatine kinase, and dependency on a wheelchair for locomotion. A gradual reduction of corticosteroids was attempted, and the patient presented with a recurrence of symptoms and renewed increase in creatine kinase levels. After further review and discussion, we decided to administer intravenous immunoglobulin; however, the patient has not yet undergone treatment due to its unavailability in the public health system.

Because of the results, a new review of the history and chronology of the events was conducted by specialists. The diagnosis of probable post-vaccination IMNM was made due to the absence of other factors that may have led to the development of autoantibodies (medicines; e.g., statins, drugs) and the temporal relationship between exposure and event.

## 3. Discussion

Idiopathic inflammatory myopathies or myositis comprises a cluster of heterogeneous autoimmune diseases characterized by muscle inflammation and distinct symptoms, including muscle weakness, myalgia, and elevated muscle enzymes [5]. The treatment relies mostly on immunosuppression, and the clinical course and treatment are variable [5]. IMNMs are distinct diseases inside this group and present with severe proximal muscle weakness, elevated muscle enzyme concentrations, myopathic findings in electroneuromyography, and necrosis in muscle biopsies [6]. Patients can be classified according to the autoantibody identified as anti-SRP, anti-HMG-CoA, or autoantibody negative [6].

Anti-HMG-CoA is the second most identified antibody associated with immune-mediated necrotizing myopathy [7]. Patients usually present subacute or chronic progressive proximal bilateral and symmetrical weakness, myalgia, dysphagia, and elevated serum creatine kinase levels [8]. It is most related to exposure to statins [9] but is also reported following infections [10–12], malignancies [13], other risk factors, and no known risk factors [8].

Vaccination is a major public health advancement but can also dysregulate autoimmune responses [14]. The COVID-19 mRNA vaccine is a lipid nanoparticle-encapsulated protein that encodes the SARS-CoV-2 spike glycoprotein [15]. This vaccine works by inducing the T-cell immune response and has been reported to induce a strong type I interferon response [16]. Recently, a review showed that most patients who develop inflammatory myopathies had received the Pfizer-BioNTech (BNT162b2) vaccine and reported symptoms just after the first dose [17].

To the best of our knowledge, this is the first case report suggesting the COVID-19 vaccine as a possible trigger for anti-HMG-CoA necrotizing myopathy. An extensive search in PubMed using the MeSH terms “myositis” and “coronavirus” and “vaccination” returned 134 results. Of these, only two reported IMNM after SARS-CoV-2 vaccination, using CoronaVac and Pfizer-BioNTech BNT162b2. Another study reported IMNM after yellow fever, tetanus/diphtheria, and hepatitis B. The studies mentioned above suggested the presence of anti-SRP antibodies, but none described the presence of anti-HMG-CoA autoantibodies (Table 1) [18–20].

## Figures and Tables

**Table 1:** Published reports of immune-mediated necrotizing myositis and previous vaccination.

Authors	Year	Age	Sex	Time from vaccination to symptom onset	Vaccine	Treatment
Cavalcanti et al. [18]	2021	42	Female	2 weeks	Yellow fever, tetanus/diphtheria, and hepatitis B	Prednisone 1 mg/kg/day
Dodig et al. [19]	2021	55	Female	21 days after 2nd dose	Pfizer-BioNTech (BNT162b2 mRNA)	Prednisone 30 mg twice; IVIG 1 g/kg; methotrexate 12.5/25 mg daily
Tan et al. [20]	2021	54	Male	1 week after 2nd dose	CoronaVac (Sinovac Biotech)	IVIG 2 g/kg followed by oral prednisolone 60 mg daily

IVIG: intravenous immunoglobulin.

## Data Availability

The clinical data used to support the findings of this study are included within the article.

## Abbreviations

IMNM: Immune-mediated necrotizing myositis
HMG-CoA: 3-hydroxy-3-methylglutaryl-coenzyme A reductase
SRP: Signal recognition particle.
